# Supplementary material for: Conceptual Model on Access to Food in the Favela Food Environment
Source: Int J Environ Res Public Health. 2024 Oct 26;21(11):1422. doi: 10.3390/ijerph21111422 (PMC11593643; doi:10.3390/ijerph21111422)
Supplement: Supplementary file 1 [file ijerph-21-01422-s001.zip › Supplementary Material S1.pdf]

Supplementary Material S1. A preliminary conceptual model on the relationships between the food environment and food access for favela residents has been proposed, consisting of a graphic scheme and a table detailing its elements.

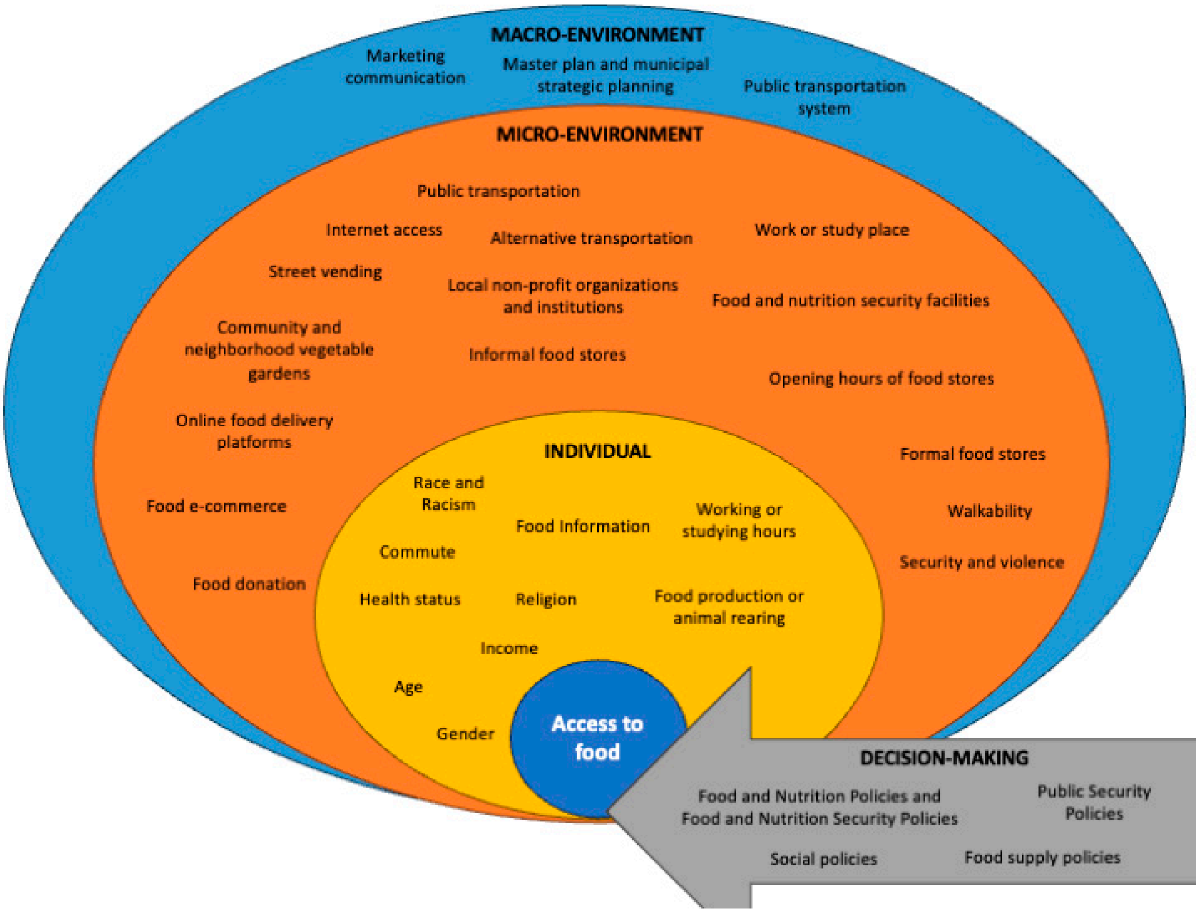

Figure 1. Preliminary version of the conceptual model on food access in Brazilian favelas.

Definitions of Dimensions:

Food Access: The concept of food access is based on the five dimensions adopted by Swinburn et al. (2013): availability, physical proximity/accessibility, financial accessibility, acceptability, and convenience. These dimensions were initially defined by Penchansky & Thomas in 1981 and later updated for the food environment domain by Caspi et al. in 2012. Availability refers to the adequacy of food supply, such as the presence and number of food purchase locations near individuals' homes or places they frequent. Physical proximity or accessibility refers to the location of food sale establishments and the ease or difficulty of reaching them, with travel time and distance being the main measures. Financial accessibility refers to food prices and individuals' perception of costs. Acceptability pertains to individuals'

attitudes toward available food items and whether these offerings align with their personal norms. Convenience refers to the acceptance and adaptation of local food sale establishments to individuals' needs, such as store hours and accepted payment methods.

Individual Level: The individual level definition is based on Story et al. (2008) concept of individual-level factors related to food choices and behaviors, referring to individual characteristics, conditions, and behaviors that can affect and influence food access.

Micro-environment: The adopted microenvironment definition was proposed by Swinburn, Egger, & Raza in 1999 and refers to a context where groups of people gather for specific purposes involving food. These contexts are generally geographically distinct, relatively small, and potentially influenced by individuals.

Macro-environment: The macroenvironment definition adopted was proposed by Swinburn, Egger, & Raza in 1999 and refers to a group of industries, services, or supporting infrastructures that influence the foods consumed in various microenvironment contexts.

Decision-making: The decision level definition was based on the concept proposed by Castro & Canella in 2022 when producing the conceptual model for the organizational food environment. In this context, decision level refers to power relations and decision-making processes that affect or interfere with the food environment, such as municipal, state, or national policies, programs, laws, and regulations.

Chart 1. Description of Elements Comprising the Conceptual Model on Food Access in Brazilian Favelas.

LEVEL	COMPONENTS	DEFINITION
Individual	Gender	Socially constructed characteristics, roles, behaviors, expectations, and identities associated with being male, female, or other gender identities such as non-binary, agender, etc.
	Age	The life stage of the individual, expressed by the amount of time elapsed since birth. Example: childhood, adolescence, senescence.
	Income	The financial importance that the individual periodically receives as remuneration for work or services rendered. Examples: salary, pension, aid, among other financial resources.

	Race and Racism	The belief that it is possible to classify humans into different naturally hierarchical races based not only on physical aspects but also on moral, psychological, intellectual, and cultural characteristics.
	Religion	A set of principles, beliefs, and practices of religious doctrines based on sacred texts that unite their followers into the same moral community.
	Working or studying hours	The daily period when the resident is available for work and/or is in class or dedicating time to studies. It also includes travel time to these locations when activities are conducted in person.
	Health Status	The health conditions of the resident that allow or prevent access to food. Examples: comorbidities that affect mobility or the development of daily activities such as obesity, chronic non-communicable diseases (NCDs), congenital and/or acquired physical and/or mental disabilities that affect or hinder mobility or the development of daily activities.
	Food production or animal rearing	The production of food and/or raising animals for personal consumption, with the possibility of selling the surplus in the region. Examples: gardens (vertical, horizontal) in backyards or balconies of residences, or raising animals for food purposes (as well as their products such as milk, honey, and eggs).
	Food Information	Data, knowledge, messages, and content related to food, nutrition, diets, eating habits, food security, and all aspects involving food intake.
	Commute	Time spent and route taken when traveling between different physical points using any means of transport.
Micro-environment	Community and neighborhood vegetable gardens	Presence of community gardens or productive backyards of neighbors or other forms of food production or animal husbandry for distribution and sale in the community. Examples: private gardens (vertical, horizontal) in the backyards or balconies of residences and/or community gardens initiated by the population or the government in public spaces.

	Food donation	Donation of food on a small or large scale by a single person, volunteer groups, or organizations and institutions, which may or may not be mediated by third sector organizations or community leaders. These donations can be made by churches, non-governmental organizations, government institutions, among other associations or individuals who donate food.
	Formal food stores	Establishments with formal fixed points that pay taxes, are inspected, and have government registrations focusing on food sales. These food sale establishments have a National Register of Legal Entities (CNPJ) or other types of registration for regulation.
	Informal food stores	Establishments with informal fixed points focusing on food sales, which may or may not be regulated and inspected by government agencies, characterized by lack of specialization, low capital investment, non-reporting, non-payment of some or all taxes, and social innovations. These food sale establishments do not have a National Register of Legal Entities (CNPJ) or other type of registration but can be registered with the National Health Surveillance Agency (Anvisa) for sanitary inspections.
	Public Food and nutrition security facilities	Equipment that integrates a set of policies and actions implemented by the state to guarantee the human right to adequate and healthy food for the entire population. These are physical structures, programs, or services offered by the government at different levels (municipal, state, or federal) to promote access to quality food, adequate nutrition, and food security. Examples: popular restaurants, open markets, food banks, public markets, community kitchens, among others.
	Street vending	The sale of food in open or closed spaces permanently with a fixed point or not. The street vendor may or may not be registered with government agencies and pay taxes, being subject to inspection. Examples: food vendors with a mobile selling space (carts, mobile

		stands, cars, bicycles, among others).
	Food e-commerce	Sale of food through online stores where all parts of the product purchase are online: product selection, choice of delivery or pick-up address, payment method, and purchase.
	Online food delivery platforms	Applications that host one or more companies that sell ready-to-eat or non-ready-to-eat food with a delivery service. They are business platforms offering order management, delivery, and payment services to food-selling establishments.
	Local non-profit organizations and institutions	Non-profit organizations that provide support and free services to favela residents.
	Internet access	Availability of broadband connection or wireless internet access points, either private or free.
	Security and violence	Perception of safety and violence experienced by residents influenced by drug trafficking, policing, confrontations, assaults, among other types of situations causing insecurity or safety to residents.
	Alternative transportation	Existence of unregulated collective and/or individual transportation means for travel within the favela. Examples: motorcycle taxis, vans, among others.
	Public transportation	Existence of accessible public transport and lines that access favelas and connect to other parts of the city.
	Work or study place	Food sold and/or provided free of charge for immediate consumption in educational institutions and workplaces located in favelas or frequented by favela residents.
	Walkability	The physical structure of the urban space that allows and encourages walking. Examples: street elevation level, existence and condition of sidewalks, sidewalk width, obstructions, public lighting, tree coverage, crosswalks,

		existence and access to bus stops, block size, among others.
Macro-environment	Marketing communication	Set of strategies, messages, and marketing practices used by companies and organizations in the food industry to promote and sell their food products. These strategies aim to influence consumer perception, increase brand recognition, and stimulate food product purchases. Examples: television and social media advertising, attractive labels with health claims, reward programs, combos, and discounts, among others.
	Master plan and municipal strategic planning	Legal and technical urban planning tool used by municipalities and municipal authorities to guide urban development and land use planning. The master plan is a document that establishes guidelines, objectives, policies, and norms for the growth and land use of a city or metropolitan region.
	Food production and distribution	Interconnected processes, activities, and infrastructures involved in food production and distribution, including food cultivation, the transformation of raw materials into food products, and the physical movement of food from production to sale points or consumers.
	Public transportation system	Infrastructure and set of organized services to enable the efficient and accessible movement of people within a city, metropolitan region, or urban area. It is designed to meet the mobility needs of urban populations.
Decision-making	Food and nutrition policies and Food and nutrition security policies	Sets of strategies, plans, programs, and actions implemented by governments and organizations to promote healthy eating, ensure adequate and sufficient access to healthy foods, and prevent malnutrition and food and nutrition insecurity. These policies are designed to address issues related to nutrition, public health, food security, and access to healthy and sustainable foods.

	Social policies	Set of actions, programs, measures, and strategies implemented by the state or other governmental and non-governmental institutions to address social issues and promote the well-being of the population. These policies aim to meet basic needs and ensure fundamental rights for individuals, especially those in situations of vulnerability or social disadvantage.
	Food supply policies	Set of strategies, regulations, governmental actions, and programs aimed at ensuring an adequate and sustainable food supply for the population of a country, region, or community. The main objective of these policies is to ensure that the population has access to safe, nutritious, and sufficient food to meet their basic dietary needs.
	Public security policies	Set of strategies, guidelines, actions, and measures implemented by governments and public institutions with the objective of promoting security, preventing crime, protecting citizens, and ensuring the enforcement of laws within a given jurisdiction. These policies are designed to address a variety of security-related challenges, ranging from crime prevention to emergency and disaster response.

References

Caspi CE, Sorensen G, Subramanian SV, Kawachi I. The local food environment and diet: a systematic review. Health Place. 2012 Sep;18(5):1172-87. doi: 10.1016/j.healthplace.2012.05.006.

Castro IRR, Canella DS. Organizational Food Environments: Advancing Their Conceptual Model. Foods. 2022 Mar 29;11(7):993. doi: 10.3390/foods11070993.

Penchansky R, Thomas JW. The concept of access: definition and relationship to consumer satisfaction. Med Care. 1981;19(2):127-40. doi: 10.1097/00005650-198102000-00001.

Story M, Kaphingst KM, Robinson-O'Brien R, Glanz K. Creating healthy food and eating environments: policy and environmental approaches. Annu Rev Public Health. 2008;29:253-72. doi: 10.1146/annurev.publhealth.29.020907.090926.

Swinburn B, Egger G, Raza F. Dissecting obesogenic environments: the development and application of a framework for identifying and prioritizing environmental interventions for obesity. Prev Med. 1999 Dec;29(6 Pt 1):563-70. doi: 10.1006/pmed.1999.0585.

Swinburn B, Sacks G, Vandevijvere S, Kumanyika S, Lobstein T, Neal B, et al. INFORMAS (International Network for Food and Obesity/non-communicable diseases Research, Monitoring and Action Support): overview and key principles. Obes Rev. 2013;14 Suppl 1:1-12. doi: 10.1111/obr.12087.
