# Supplementary material for: Conceptual Model on Access to Food in the Favela Food Environment
Source: Int J Environ Res Public Health. 2024 Oct 26;21(11):1422. doi: 10.3390/ijerph21111422 (PMC11593643; doi:10.3390/ijerph21111422)
Supplement: Supplementary file 1 [file ijerph-21-01422-s001.zip › Supplementary Material S2.pdf]

Supplementary Material S2. Online form sent to experts for the evaluation of the conceptual model and table of definitions.

Conceptual Model of the Food Environment in Favelas

Dear participants, we appreciate your availability to participate in the expert panel to evaluate the conceptual model of the food environment in favelas.

In this panel, you will evaluate the conceptual model and the table of definitions of the dimensions and variables included in the model.

Below each question, there is a space for comments if you choose an option other than "strongly agree."

Before starting to fill out each section of the form, read all the questions to better understand their nature and the time required.

The average time to complete this form is 20 minutes.

We recommend that you read the explanatory document of the model sent previously to your email before starting to fill out this form.

Feel free to evaluate the model and leave your contributions and impressions!

* Indicates a required question

1. Full Name (Important for issuing certification!)*
2. Email*
3. City/State*
4. Organization/Institution*
5. Gender (Select only one option)*
    - Female
    - Male
    - Non-binary
    - Prefer not to say
    - Other:
6. Race/Color (Select only one option)*
    - White
    - Black
    - Brown

- Yellow
- Indigenous
- Prefer not to say
- Other:

Informed Consent Form (ICF)

We invite you to voluntarily participate in the expert panel linked to the research project "Food Environment of Villages and Favelas in a Brazilian Metropolis."

The aim of this research is to evaluate the food environment of favelas in a Brazilian metropolis. Through this project, we hope to expand knowledge about the food environment in favelas. Additionally, the main idea is to generate support for the implementation of public policies on food and nutritional security, as well as to optimize the interdisciplinarity of actions by different professionals working in these territories to reduce food inequalities.

This panel aims to evaluate the conceptual model of the food environment in favelas. You will receive all necessary clarifications before and during the research regarding the expected activities arising from your participation, as well as the materials to be evaluated. The participation method in this panel will be through an evaluation form of the model and its adopted definitions.

To participate in this research, we need you to answer some questions. The time spent filling out the form is approximately 20 minutes. We will treat your identity with professional confidentiality standards. The risks resulting from your participation in the research are limited to potential discomfort if any question causes unease in filling out the form and maintaining the confidentiality of your identity and the information you provide. However, all precautions will be taken to preserve your identity. We emphasize that the purpose of the research is not to evaluate your work processes or measure your technical capacity but to understand, from your personal perspective and expertise, the best model to explain the food environment in favelas.

Your name will be kept confidential, and your responses will be used solely for this research.

You may refuse to answer any question that causes discomfort and may withdraw from participating in the research at any time without any penalty. Your participation is voluntary, meaning there is no payment for your collaboration.

There will be no costs on your part for participating in this research.

You are guaranteed to receive answers to any questions and clarification of any doubts regarding the research. Additionally, we, the researchers, commit to providing updated information obtained during the study.

If there is any direct or indirect harm resulting from your participation in the research, you should seek compensation following the legal provisions in force in Brazil.

The research results will be disseminated in the academic processes of the Federal University of Minas Gerais and may be published later. The data and materials will be used solely for this research and will be kept by the researcher for five years, after which they will be destroyed.

If you have any questions regarding the research, please contact: xxxxxxxxxxxxxx, available for WhatsApp messages as well, or via email at xxxxxxxxxxxx.

This project has been approved by the Research Ethics Committee (REC) of the Federal University of Minas Gerais. The REC comprises professionals from different fields whose function is to defend the interests of research participants in their integrity and dignity and to contribute to the development of research within ethical standards. Questions regarding the signing of the ICF or the participant's rights can be clarified by phone at xxxxxxxx or by email at xxxxxxxxx, with service hours from 9:00 am to 11:00 am and 2:00 pm to 4:00 pm, Monday to Friday. The REC is located at xxxxxxxxxxxxxx.

If you agree to participate, please select the option "Yes, and I agree to participate in the research" in the box below.

7. Given the explanations, do you feel sufficiently informed about the research to be conducted and agree to voluntarily participate as a collaborator? (Select only one option)*
   - No, and I do not agree to participate in the research
   - Yes, and I agree to participate in the research

Conceptual Model of the Food Environment in Favelas

Graphic scheme of the Conceptual Model of the Food Environment in Favelas

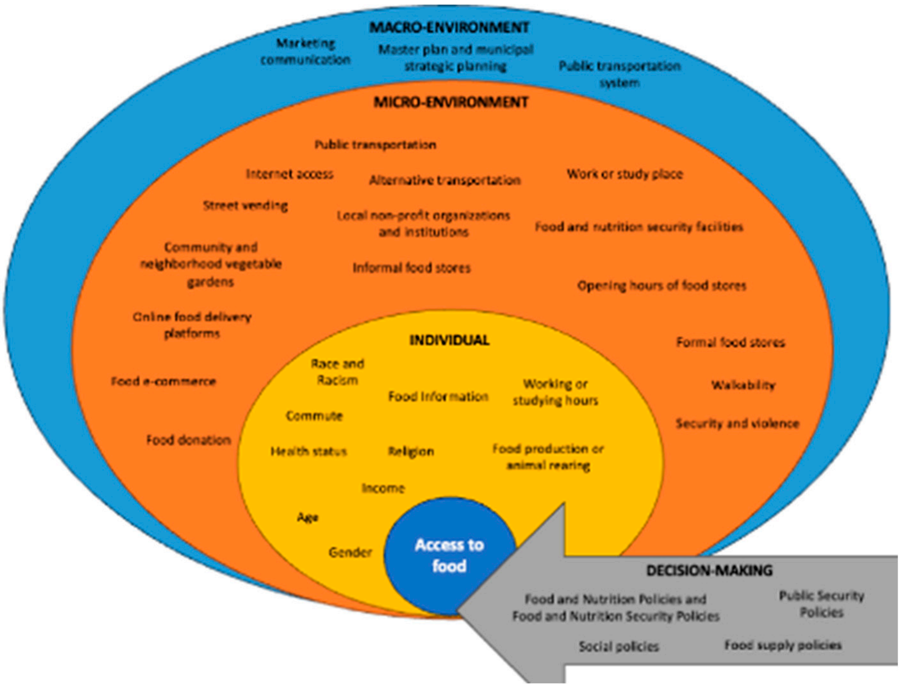

Table of Definitions - FOOD ACCESS

The concept of food access is based on the five dimensions adopted by Swinburn et al. (2013): availability, physical proximity/accessibility, financial accessibility, acceptability, and convenience. These dimensions were initially defined by Penchansky & Thomas in 1981 and later updated for the food environment domain by Caspi et al. in 2012. Availability refers to the adequacy of food supply, such as the presence and number of food purchase locations near individuals' homes or places they frequent. Physical proximity or accessibility refers to the location of food sale establishments and the ease or difficulty of reaching them, with travel time and distance being the main measures. Financial accessibility refers to food prices and individuals' perception of costs. Acceptability pertains to individuals' attitudes toward available food items and whether these offerings align with their personal norms. Convenience refers to the acceptance and adaptation of local food sale establishments to individuals' needs, such as store hours and accepted payment methods.

Table of Definitions of the Dimensions Included in the Model

Individual Level: The individual level definition is based on Story et al. (2008) concept of individual-level factors related to food choices and behaviors, referring to individual characteristics, conditions, and behaviors that can affect and influence food access.

Micro-environment: The adopted microenvironment definition was proposed by Swinburn, Egger, & Raza in 1999 and refers to a context where groups of people gather for specific

purposes involving food. These contexts are generally geographically distinct, relatively small, and potentially influenced by individuals.

Macro-environment: The macroenvironment definition adopted was proposed by Swinburn, Egger, & Raza in 1999 and refers to a group of industries, services, or supporting infrastructures that influence the foods consumed in various microenvironment contexts.

Decision-making: The decision level definition was based on the concept proposed by Castro & Canella in 2022 when producing the conceptual model for the organizational food environment. In this context, decision level refers to power relations and decision-making processes that affect or interfere with the food environment, such as municipal, state, or national policies, programs, laws, and regulations.

Table of Definitions of the Variables Included in the Model

<u>Individual</u>

Gender

Socially constructed characteristics, roles, behaviors, expectations, and identities associated with being male, female, or other gender identities such as non-binary, agender, etc.

Age

The life stage of the individual, expressed by the amount of time elapsed since birth. Example: childhood, adolescence, senescence.

Income

The financial importance that the individual periodically receives as remuneration for work or services rendered. Examples: salary, pension, aid, among other financial resources.

Race and Racism

The belief that it is possible to classify humans into different naturally hierarchical races based not only on physical aspects but also on moral, psychological, intellectual, and cultural characteristics.

Religion

A set of principles, beliefs, and practices of religious doctrines based on sacred texts that unite their followers into the same moral community.

Working or studying hours

The daily period when the resident is available for work and/or is in class or dedicating time to studies. It also includes travel time to these locations when activities are conducted in person.

Health Status

The health conditions of the resident that allow or prevent access to food. Examples: comorbidities that affect mobility or the development of daily activities such as obesity, chronic non-communicable diseases (NCDs), congenital and/or acquired physical and/or mental disabilities that affect or hinder mobility or the development of daily activities.

Food production or animal rearing

The production of food and/or raising animals for personal consumption, with the possibility of selling the surplus in the region. Examples: gardens (vertical, horizontal) in backyards or balconies of residences, or raising animals for food purposes (as well as their products such as milk, honey, and eggs).

Food Information

Data, knowledge, messages, and content related to food, nutrition, diets, eating habits, food security, and all aspects involving food intake.

Commute

Time spent and route taken when traveling between different physical points using any means of transport.

Micro-environment

Community and neighborhood vegetable gardens

Presence of community gardens or productive backyards of neighbors or other forms of food production or animal husbandry for distribution and sale in the community. Examples: private gardens (vertical, horizontal) in the backyards or balconies of residences and/or community gardens initiated by the population or the government in public spaces.

Food donation

Donation of food on a small or large scale by a single person, volunteer groups, or organizations and institutions, which may or may not be mediated by third sector organizations or community

leaders. These donations can be made by churches, non-governmental organizations, government institutions, among other associations or individuals who donate food.

Formal food stores

Establishments with formal fixed points that pay taxes, are inspected, and have government registrations focusing on food sales. These food sale establishments have a National Register of Legal Entities (CNPJ) or other types of registration for regulation.

Informal food stores

Establishments with informal fixed points focusing on food sales, which may or may not be regulated and inspected by government agencies, characterized by lack of specialization, low capital investment, non-reporting, non-payment of some or all taxes, and social innovations. These food sale establishments do not have a National Register of Legal Entities (CNPJ) or other type of registration but can be registered with the National Health Surveillance Agency (Anvisa) for sanitary inspections.

Public Food and nutrition security facilities

Equipment that integrates a set of policies and actions implemented by the state to guarantee the human right to adequate and healthy food for the entire population. These are physical structures, programs, or services offered by the government at different levels (municipal, state, or federal) to promote access to quality food, adequate nutrition, and food security. Examples: popular restaurants, open markets, food banks, public markets, community kitchens, among others.

Street vending

The sale of food in open or closed spaces permanently with a fixed point or not. The street vendor may or may not be registered with government agencies and pay taxes, being subject to inspection. Examples: food vendors with a mobile selling space (carts, mobile stands, cars, bicycles, among others).

Food e-commerce

Sale of food through online stores where all parts of the product purchase are online: product selection, choice of delivery or pick-up address, payment method, and purchase.

Online food delivery platforms

Applications that host one or more companies that sell ready-to-eat or non-ready-to-eat food with a delivery service. They are business platforms offering order management, delivery, and payment services to food-selling establishments.

Local non-profit organizations and institutions

Non-profit organizations that provide support and free services to favela residents.

Internet access

Availability of broadband connection or wireless internet access points, either private or free.

Security and violence

Perception of safety and violence experienced by residents influenced by drug trafficking, policing, confrontations, assaults, among other types of situations causing insecurity or safety to residents.

Alternative transportation

Existence of unregulated collective and/or individual transportation means for travel within the favela. Examples: motorcycle taxis, vans, among others.

Public transportation

Existence of accessible public transport and lines that access favelas and connect to other parts of the city.

Work or study place

Food sold and/or provided free of charge for immediate consumption in educational institutions and workplaces located in favelas or frequented by favela residents.

Walkability

The physical structure of the urban space that allows and encourages walking. Examples: street elevation level, existence and condition of sidewalks, sidewalk width, obstructions, public lighting, tree coverage, crosswalks, existence and access to bus stops, block size, among others.

Macro-environment

Marketing communication

Set of strategies, messages, and marketing practices used by companies and organizations in the food industry to promote and sell their food products. These strategies aim to influence consumer perception, increase brand recognition, and stimulate food product purchases. Examples: television and social media advertising, attractive labels with health claims, reward programs, combos, and discounts, among others.

Master plan and municipal strategic planning

Legal and technical urban planning tool used by municipalities and municipal authorities to guide urban development and land use planning. The master plan is a document that establishes guidelines, objectives, policies, and norms for the growth and land use of a city or metropolitan region.

Food production and distribution

Interconnected processes, activities, and infrastructures involved in food production and distribution, including food cultivation, the transformation of raw materials into food products, and the physical movement of food from production to sale points or consumers.

Public transportation system

Infrastructure and set of organized services to enable the efficient and accessible movement of people within a city, metropolitan region, or urban area. It is designed to meet the mobility needs of urban populations.

Decision-making

Food and nutrition policies and Food and nutrition security policies

Sets of strategies, plans, programs, and actions implemented by governments and organizations to promote healthy eating, ensure adequate and sufficient access to healthy foods, and prevent malnutrition and food and nutrition insecurity. These policies are designed to address issues related to nutrition, public health, food security, and access to healthy and sustainable foods.

Social policies

Set of actions, programs, measures, and strategies implemented by the state or other governmental and non-governmental institutions to address social issues and promote the well-being of the population. These policies aim to meet basic needs and ensure fundamental rights for individuals, especially those in situations of vulnerability or social disadvantage.

Food supply policies

Set of strategies, regulations, governmental actions, and programs aimed at ensuring an adequate and sustainable food supply for the population of a country, region, or community. The main objective of these policies is to ensure that the population has access to safe, nutritious, and sufficient food to meet their basic dietary needs.

Public security policies

Set of strategies, guidelines, actions, and measures implemented by governments and public institutions with the objective of promoting security, preventing crime, protecting citizens, and ensuring the enforcement of laws within a given jurisdiction. These policies are designed to address a variety of security-related challenges, ranging from crime prevention to emergency and disaster response.

EVALUATION - GRAPHIC SCHEME

After consulting with experts, the graphic scheme of the model will be visually enhanced with the support of a graphic designer.

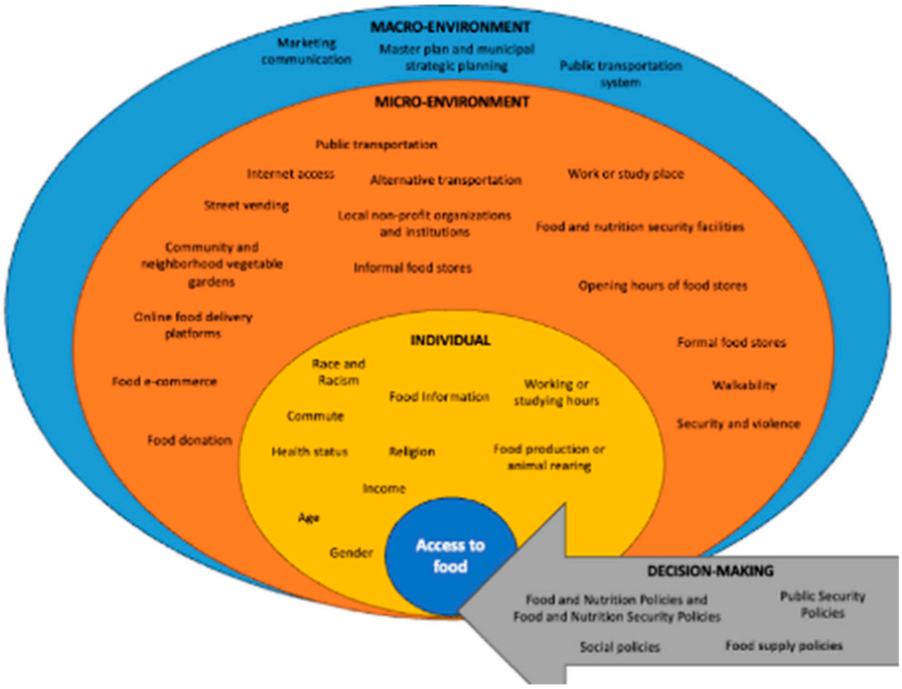

8. All CONSTITUTIVE ELEMENTS included are RELEVANT to the food environment of favelas. (Select only one option)*
    - Strongly agree
    - Partially agree

- Neither agree nor disagree
- Partially disagree
- Strongly disagree

9. Comments

   Space for comments if you choose an option other than "strongly agree."

10. The CONSTITUTIVE ELEMENTS included in the model COVER ALL RELEVANT COMPONENTS for the food environment of favelas. (Select only one option)*
    - Strongly agree
    - Partially agree
    - Neither agree nor disagree
    - Partially disagree
    - Strongly disagree

11. Comments

    Space for comments if you choose an option other than "strongly agree."

12. The conceptual model IS CLEAR. (Select only one option)*
    - Strongly agree
    - Partially agree
    - Neither agree nor disagree
    - Partially disagree
    - Strongly disagree

13. Comments

    Space for comments if you choose an option other than "strongly agree."

14. The FORMAT of the GRAPHIC REPRESENTATION of the conceptual model is CLEAR. (Select only one option)*
    - Strongly agree
    - Partially agree
    - Neither agree nor disagree
    - Partially disagree
    - Strongly disagree

15. Comments

    Space for comments if you choose an option other than "strongly agree."

16. The TERMS used to name the constitutive elements of the GRAPHIC REPRESENTATION of the conceptual model are CLEAR. (Select only one option)*
    - Strongly agree
    - Partially agree
    - Neither agree nor disagree
    - Partially disagree
    - Strongly disagree
17. Comments

    Space for comments if you choose an option other than "strongly agree."
18. Space for general comments on the graphic representation of the conceptual model for the food environment of favelas.

EVALUATION - CONCEPT OF FOOD ACCESS

The concept of food access is based on the five dimensions adopted by Swinburn et al. (2013): availability, physical proximity/accessibility, financial accessibility, acceptability, and convenience. These dimensions were initially defined by Penchansky & Thomas in 1981 and later updated for the food environment domain by Caspi et al. in 2012. Availability refers to the adequacy of food supply, such as the presence and number of food purchase locations near individuals' homes or places they frequent. Physical proximity or accessibility refers to the location of food sale establishments and the ease or difficulty of reaching them, with travel time and distance being the main measures. Financial accessibility refers to food prices and individuals' perception of costs. Acceptability pertains to individuals' attitudes toward available food items and whether these offerings align with their personal norms. Convenience refers to the acceptance and adaptation of local food sale establishments to individuals' needs, such as store hours and accepted payment methods.

19. The description of food access is easy to understand. (Select only one option)*
    - Strongly agree
    - Partially agree
    - Neither agree nor disagree
    - Partially disagree
    - Strongly disagree
20. Comments

    Space for comments if you choose an option other than "strongly agree."

EVALUATION - DIMENSIONS

Individual Level: The individual level definition is based on Story et al. (2008) concept of individual-level factors related to food choices and behaviors, referring to individual characteristics, conditions, and behaviors that can affect and influence food access.

Micro-environment: The adopted microenvironment definition was proposed by Swinburn, Egger, & Raza in 1999 and refers to a context where groups of people gather for specific purposes involving food. These contexts are generally geographically distinct, relatively small, and potentially influenced by individuals.

Macro-environment: The macroenvironment definition adopted was proposed by Swinburn, Egger, & Raza in 1999 and refers to a group of industries, services, or supporting infrastructures that influence the foods consumed in various microenvironment contexts.

Decision-making: The decision level definition was based on the concept proposed by Castro & Canella in 2022 when producing the conceptual model for the organizational food environment. In this context, decision level refers to power relations and decision-making processes that affect or interfere with the food environment, such as municipal, state, or national policies, programs, laws, and regulations.

21. All the DIMENSIONS included are relevant to the food environment in slums. (Select only one option)*
    - Strongly agree
    - Partially agree
    - Neither agree nor disagree
    - Partially disagree
    - Strongly disagree
22. Comments

    Space for comments if you choose an option other than "strongly agree."
23. All the DIMENSIONS that should make up a model for the food environment in slums are included in the framework. (Select only one option)*
    - Strongly agree
    - Partially agree
    - Neither agree nor disagree

- Partially disagree
- Strongly disagree

24. Comments

    Space for comments if you choose an option other than "strongly agree."

25. The set of DIMENSIONS is easy to understand. (Select only one option)*
    - Strongly agree
    - Partially agree
    - Neither agree nor disagree
    - Partially disagree
    - Strongly disagree
26. Comments

    Space for comments if you choose an option other than "strongly agree."

27. The description of the DIMENSIONS is easy to understand. (Select only one option)*
    - Strongly agree
    - Partially agree
    - Neither agree nor disagree
    - Partially disagree
    - Strongly disagree
28. Comments

    Space for comments if you choose an option other than "strongly agree."

29. Space for general comments on the framework of dimensions for the food environment of favelas

EVALUATION – VARIABLES

<u>Individual</u>

Gender

Socially constructed characteristics, roles, behaviors, expectations, and identities associated with being male, female, or other gender identities such as non-binary, agender, etc.

Age

The life stage of the individual, expressed by the amount of time elapsed since birth. Example: childhood, adolescence, senescence.

Income

The financial importance that the individual periodically receives as remuneration for work or services rendered. Examples: salary, pension, aid, among other financial resources.

Race and Racism

The belief that it is possible to classify humans into different naturally hierarchical races based not only on physical aspects but also on moral, psychological, intellectual, and cultural characteristics.

Religion

A set of principles, beliefs, and practices of religious doctrines based on sacred texts that unite their followers into the same moral community.

Working or studying hours

The daily period when the resident is available for work and/or is in class or dedicating time to studies. It also includes travel time to these locations when activities are conducted in person.

Health Status

The health conditions of the resident that allow or prevent access to food. Examples: comorbidities that affect mobility or the development of daily activities such as obesity, chronic non-communicable diseases (NCDs), congenital and/or acquired physical and/or mental disabilities that affect or hinder mobility or the development of daily activities.

Food production or animal rearing

The production of food and/or raising animals for personal consumption, with the possibility of selling the surplus in the region. Examples: gardens (vertical, horizontal) in backyards or balconies of residences, or raising animals for food purposes (as well as their products such as milk, honey, and eggs).

Food Information

Data, knowledge, messages, and content related to food, nutrition, diets, eating habits, food security, and all aspects involving food intake.

Commute

Time spent and route taken when traveling between different physical points using any means of transport.

Micro-environment

Community and neighborhood vegetable gardens

Presence of community gardens or productive backyards of neighbors or other forms of food production or animal husbandry for distribution and sale in the community. Examples: private gardens (vertical, horizontal) in the backyards or balconies of residences and/or community gardens initiated by the population or the government in public spaces.

Food donation

Donation of food on a small or large scale by a single person, volunteer groups, or organizations and institutions, which may or may not be mediated by third sector organizations or community leaders. These donations can be made by churches, non-governmental organizations, government institutions, among other associations or individuals who donate food.

Formal food stores

Establishments with formal fixed points that pay taxes, are inspected, and have government registrations focusing on food sales. These food sale establishments have a National Register of Legal Entities (CNPJ) or other types of registration for regulation.

Informal food stores

Establishments with informal fixed points focusing on food sales, which may or may not be regulated and inspected by government agencies, characterized by lack of specialization, low capital investment, non-reporting, non-payment of some or all taxes, and social innovations. These food sale establishments do not have a National Register of Legal Entities (CNPJ) or other type of registration but can be registered with the National Health Surveillance Agency (Anvisa) for sanitary inspections.

Public Food and nutrition security facilities

Equipment that integrates a set of policies and actions implemented by the state to guarantee the human right to adequate and healthy food for the entire population. These are physical structures, programs, or services offered by the government at different levels (municipal, state,

or federal) to promote access to quality food, adequate nutrition, and food security. Examples: popular restaurants, open markets, food banks, public markets, community kitchens, among others.

Street vending

The sale of food in open or closed spaces permanently with a fixed point or not. The street vendor may or may not be registered with government agencies and pay taxes, being subject to inspection. Examples: food vendors with a mobile selling space (carts, mobile stands, cars, bicycles, among others).

Food e-commerce

Sale of food through online stores where all parts of the product purchase are online: product selection, choice of delivery or pick-up address, payment method, and purchase.

Online food delivery platforms

Applications that host one or more companies that sell ready-to-eat or non-ready-to-eat food with a delivery service. They are business platforms offering order management, delivery, and payment services to food-selling establishments.

Local non-profit organizations and institutions

Non-profit organizations that provide support and free services to favela residents.

Internet access

Availability of broadband connection or wireless internet access points, either private or free.

Security and violence

Perception of safety and violence experienced by residents influenced by drug trafficking, policing, confrontations, assaults, among other types of situations causing insecurity or safety to residents.

Alternative transportation

Existence of unregulated collective and/or individual transportation means for travel within the favela. Examples: motorcycle taxis, vans, among others.

Public transportation

Existence of accessible public transport and lines that access favelas and connect to other parts of the city.

Work or study place

Food sold and/or provided free of charge for immediate consumption in educational institutions and workplaces located in favelas or frequented by favela residents.

Walkability

The physical structure of the urban space that allows and encourages walking. Examples: street elevation level, existence and condition of sidewalks, sidewalk width, obstructions, public lighting, tree coverage, crosswalks, existence and access to bus stops, block size, among others.

Macro-environment

Marketing communication

Set of strategies, messages, and marketing practices used by companies and organizations in the food industry to promote and sell their food products. These strategies aim to influence consumer perception, increase brand recognition, and stimulate food product purchases. Examples: television and social media advertising, attractive labels with health claims, reward programs, combos, and discounts, among others.

Master plan and municipal strategic planning

Legal and technical urban planning tool used by municipalities and municipal authorities to guide urban development and land use planning. The master plan is a document that establishes guidelines, objectives, policies, and norms for the growth and land use of a city or metropolitan region.

Food production and distribution

Interconnected processes, activities, and infrastructures involved in food production and distribution, including food cultivation, the transformation of raw materials into food products, and the physical movement of food from production to sale points or consumers.

Public transportation system

Infrastructure and set of organized services to enable the efficient and accessible movement of people within a city, metropolitan region, or urban area. It is designed to meet the mobility needs of urban populations.

Decision-making

Food and nutrition policies and Food and nutrition security policies

Sets of strategies, plans, programs, and actions implemented by governments and organizations to promote healthy eating, ensure adequate and sufficient access to healthy foods, and prevent malnutrition and food and nutrition insecurity. These policies are designed to address issues related to nutrition, public health, food security, and access to healthy and sustainable foods.

Social policies

Set of actions, programs, measures, and strategies implemented by the state or other governmental and non-governmental institutions to address social issues and promote the well-being of the population. These policies aim to meet basic needs and ensure fundamental rights for individuals, especially those in situations of vulnerability or social disadvantage.

Food supply policies

Set of strategies, regulations, governmental actions, and programs aimed at ensuring an adequate and sustainable food supply for the population of a country, region, or community. The main objective of these policies is to ensure that the population has access to safe, nutritious, and sufficient food to meet their basic dietary needs.

Public security policies

Set of strategies, guidelines, actions, and measures implemented by governments and public institutions with the objective of promoting security, preventing crime, protecting citizens, and ensuring the enforcement of laws within a given jurisdiction. These policies are designed to address a variety of security-related challenges, ranging from crime prevention to emergency and disaster response.

30. All the VARIABLES included are relevant to the food environment in slums. (Select only one option)*
    - Strongly agree
    - Partially agree
    - Neither agree nor disagree
    - Partially disagree
    - Strongly disagree
31. Comments

Space for comments if you choose an option other than "strongly agree."

32. All the VARIABLES that should make up a model for the food environment in slums are included in the framework. (Select only one option)*
    - Strongly agree
    - Partially agree
    - Neither agree nor disagree
    - Partially disagree
    - Strongly disagree
33. Comments

    Space for comments if you choose an option other than "strongly agree."
34. The set of VARIABLES is easy to understand. (Select only one option)*
    - Strongly agree
    - Partially agree
    - Neither agree nor disagree
    - Partially disagree
    - Strongly disagree
35. Comments

    Space for comments if you choose an option other than "strongly agree."
36. The description of the VARIABLES is easy to understand. (Select only one option)*
    - Strongly agree
    - Partially agree
    - Neither agree nor disagree
    - Partially disagree
    - Strongly disagree
37. Comments

    Space for comments if you choose an option other than "strongly agree."
38. Space for general comments on the framework of variables for the food environment of favelas

ACKNOWLEDGEMENTS

The empirical material collected from the expert panel will be examined by the researchers considering the adopted framework, and suggestions that contribute to the improvement and development of the final version of the conceptual model will be incorporated.

We appreciate your participation and the sharing of your insights.

Soon, you will receive the certificate of participation in the expert panel at the email address provided in this form.

Best regards,
