# Supplementary material for: Conceptual Model on Access to Food in the Favela Food Environment
Source: Int J Environ Res Public Health. 2024 Oct 26;21(11):1422. doi: 10.3390/ijerph21111422 (PMC11593643; doi:10.3390/ijerph21111422)
Supplement: Supplementary file 1 [file ijerph-21-01422-s001.zip › Supplementary Material S3.pdf]

Supplementary material S3. Systematization of the suggestions made by the panel of experts.

DESFECTO	DEFINITION	Suggestions	Answer
Food Access	The concept of food access is based on the five dimensions adopted by Swinburn et al. (2013): availability, physical proximity/accessibility, financial accessibility, acceptability, and convenience. These dimensions were initially defined by Penchansky & Thomas in 1981 and later updated for the food environment domain by Caspi et al. in 2012. Availability refers to the adequacy of food supply, such as the presence and number of food purchase locations near individuals' homes or places they frequent. Physical proximity or accessibility refers to the location of food sale establishments and the ease or difficulty of reaching them, with travel time and distance being the main measures. Financial accessibility refers to food prices and individuals' perception of costs. Acceptability pertains to individuals' attitudes toward available food items and whether these offerings align with their personal norms. Convenience refers to the acceptance and adaptation of local food sale establishments to individuals' needs, such as store hours and accepted payment methods.	The definition has many elements that can get lost, an alternative is to put all of them in the model, for example: Availability, price, physical access, financial access, acceptability and convenience.	The term food access adopted in this model considered the dimensions of the manuscript by Caspi et al. [17] in their study on the outcome of access to food: availability, physical proximity/accessibility, financial accessibility, acceptability, and convenience. Thus, we consider that by including the term access to food as the main outcome, we are considering all aspects of the concept.
		The "individual's decision" is not a determining factor in food choices, with the diner being at the mercy of the formation of their taste by the social conventions in force where they are inserted, dictated by their exposure to the media, their class position and their insertion in the geography of the city.	We considered all the aspects, from the individual level to the level of structuring/regulating public policies, that can interfere with access to food for favela dwellers.

Individual Level	The individual level definition is based on Story et al. (2008) concept of individual-level factors related to food choices and behaviors, referring to individual characteristics, conditions, and behaviors that can affect and influence food access.	I have a few observations, as I worked as a nutritionist on an extension project in a community here in Fortaleza, called Morro do Santiago, and I traveled around the area because we were doing home visits, I noticed that it was very common for people to put up a little sign saying that they were selling snacks, plates of food, dindim (chup-chup for us miners) and I wondered if it fit in with street vendors or informal food outlets. There was also a lot of exchange of food items between neighbors and family members from other households, which I missed. Also, the number of members in the household, which has a big impact on the purchase of different types of food for the individual/family budget. I understood that income is a substitute for education. Local and third sector organizations and institutions, food donations and religion can overlap. Because it was also common for churchgoers to receive weekly/monthly donations. Criminal factions (which also don't fall under Local and Third Sector Organizations and Institutions) also donate food, as absurd as that may sound. There is a local "law" for everything that happens in the communities and favelas. It was another aspect that we organized the distribution of	With regard to the exchange of food items, we added a variable to the model at the micro-environment level, "Non-monetary food acquisition", which refers to the exchange of food items and food donations received from any source. We would point out that local non-profit organizations and institutions play different roles in the lives of people living in favelas, apart from donating food. With regard to the number of people in the household, we added a variable to the model at the individual level, "Family structure", which refers to the size of the family and the composition of those living in the household. In relation to income and schooling, we added a variable to the model at the individual level "Socioeconomic status", which considers the issues of income (of any kind, salary and benefits/aid) and schooling (which can be understood as a *proxy for* income). And to include the role of criminal factions and militias in the lives of favela residents, we included criminal organizations at the micro-environment level in the model through the variable "Other Organizations", which considers any other type of organization that may have some influence and openness in the favelas.

		this food, and even that "deserved/could" receive these donations.	
		At the individual level, I also thought about the issue of food culture, which cuts across race/ethnicity and the other obstacles that the food guide poses, such as cooking skills, space to produce, store and serve meals at home. Also at the individual level, being a beneficiary of social programs can make a difference - even though social benefits are at the decision-making level, there is a clear difference between those who have and those who don't in terms of access to food.	We added the variable "Food Culture" to the model at the individual level, which considers a set of ideas, beliefs, knowledge and practices transmitted over time, related to food and shared between and within groups. In relation to social benefits, the variable "Socioeconomic status" takes into account income (of any kind, salary and benefits) and schooling. We also included the variable "Household structure" at the individual level, which considers the available materials that people have at home to store and prepare food.
		I had doubts about whether aspects related to regional eating habits would be included in the model. For example, a favela located in Rio de Janeiro may have a significant number of migrants from the northern region of the country, and the eating habits	We added to the model the variable "Food Culture" at the individual level, which considers a set of ideas, beliefs, knowledge and practices transmitted over time, related to food and shared between and within groups.

		of this population may be related to their food choices within the favelas.	
		I suggest including "education" and "culture" at the individual level.	With regard to schooling, we added a variable to the model at the individual level, "Socioeconomic status", which takes into account income (of any kind, salary and benefits) and schooling. And we added the variable "Food Culture" to the model at the individual level, which considers a set of ideas, beliefs, knowledge and practices transmitted over time, related to food and shared between and within groups.
		Is schooling an important individual factor? Or even marital status, number of individuals in the family? The family's food security situation?	With regard to schooling, we added a variable to the model at the individual level "Socioeconomic status", which takes into account income (of any kind, salary and benefits) and schooling. Regarding the number of people in the household and marital status, we added a variable to the model at the individual level "Family structure", which refers to the size of the family and the composition of those living in the household. Regarding the situation of food and nutritional security, we consider that this is an aspect that results from the lack of access to food, considering all of its dimensions, so it is not represented in this model as a factor that modulates access to food.

		I think it's pertinent to make a substitution at the individual level - changing information about food and/or eating to access to quality education and knowledge, because this is individually what will affect the understanding of the information received and consequently habits and appropriate and healthy choices. Thus, the element of information should be included in the micro field as factors that influence access to adequate and healthy food. And the item food production in the individual field, in the conceptual model, should be specified that it is basically for own consumption, subsistence, otherwise it seems that it is the food system as a whole.	In relation to information about food and/or eating, we added the variable "Food literacy" at the individual level, which refers to the resident's ability to obtain, process and understand information about nutrition, and included the variable "Food Information" at the micro-environment level, which refers to the information available in the environment about food, such as nutritional tables and lists of ingredients, food advertising, nutritional claims, among other information. The variable on "Food production or animal rearing" considers the food that families living in favelas produce for their own consumption, barter and the sale of surpluses (which can help with the family's income and, consequently, the purchase of food).
		Particularly in the case of religion, in addition to being an individual element, its weight varies greatly depending on its incidence, power in the neighborhood or region... (if it's a group of African origin, with strong territorial ties, its influence will be different from a more specific religious link)...	We included the variable "Religion" in the model because of its importance in the lives of families living in favelas and, consequently, in food consumption (restriction of certain types of meat, for example). In addition, the religious community can help with the donation and sharing of food. The impact can vary according to the religion the family adheres to, and the model adapts to these changes, and can be more or less complex, depending on the dynamics of the favela being studied.

		It may be interesting to consider schooling, as it can influence the item "information about food and eating"	With regard to schooling, we added a variable to the model at the individual level, "Socioeconomic status", which takes into account income (of any kind, salary and benefits) and schooling.
		I also thought that "displacement" would be more interesting to remove from the individual level and keep in the microenvironment, along with "walkability" (check the use of this term in Portuguese). Still at the individual level, I suggest presenting "information and/or knowledge about food", to consider the element in a broader way.	In relation to information about food and/or eating, we added the variable "Food literacy" at the individual level, which refers to the resident's ability to obtain, process and understand information about nutrition, and we included the variable "Food Information" at the micro-environment level, which refers to the information available in the environment about food, such as nutritional tables and ingredient lists, food advertising, nutritional claims, among other information. Walkability is defined by the Institute for Transport Policy and Development as the extent to which the characteristics of the urban environment favor its use for walking. Commuting refers to the individual dynamics that favela residents need to organize and plan in order to move around the favela and the city, considering commuting to work, study, shopping, social life, among others. This commute can be on foot, using personal, alternative or public transport, depending on each individual's routine and organization, as well as the availability of alternative or public

			transport in the micro-environment.
		I suggest including education	With regard to schooling, we added a variable to the model at the individual level, "Socioeconomic status", which takes into account income (of any kind, salary and benefits) and schooling.
		As per previous comments, I suggested including "education" and "culture" at the individual level	We added the variable "Food Culture" to the model at the individual level, which considers a set of ideas, beliefs, knowledge and practices transmitted over time, related to food and shared between and within groups. In relation to schooling, we added a variable to the model at the individual level, "Socioeconomic status", which takes into account income (of any kind, salary and benefits) and schooling.
		At the individual level, the schooling variable could be included.	With regard to schooling, we added a variable to the model at the individual level, "Socioeconomic status", which takes into

			account income (of any kind, salary and benefits) and schooling.
		When we think about individual questions of acceptability, don't food preferences, cultural issues, myths and taboos come into play?	We added to the model the variable "Food Culture" at the individual level, which considers a set of ideas, beliefs, knowledge and practices transmitted over time, related to food and shared between and within groups.
		It might also be interesting to consider access to basic rights, such as water, sewage, energy and household waste collection.	We added the "Basic sanitation" variable to the model, which considers access to basic services such as drinking water distribution, sewage collection and treatment, urban drainage and solid waste collection.
		The economic issue is missing. The price of food. The salary of a father or mother to feed their family. Many people look for cheap food or can't afford to buy enough to meet their needs. After all, food is a commodity, and the economic crisis raises food prices and hunger skyrockets. This happens in the favelas.	The concept of access to food that we adopted in the model considers: availability, physical proximity/accessibility, financial accessibility, acceptability, and convenience. Thus, the price of food is being considered in the model's outcome variable.
		All the elements presented are important to consider in the model, but I believe that an important factor should be added to the individual when debating the issue of food: the number of people living in the same space.	With regard to the number of people in the household, we added a variable to the model at the individual level, "Family structure", which refers to the size of the family and the composition of those living in the household.
Micro-environment	The adopted microenvironment definition was proposed by Swinburn, Egger, & Raza in 1999 and refers to a	I think that in the micro-environment, just as the transportation system was	We added the variable "Health Services" to the model in the microenvironment, which

Level	context where groups of people gather for specific purposes involving food. These contexts are generally geographically distinct, relatively small, and potentially influenced by individuals.	considered, I believe that for the favelas the local health system also makes a big difference in promoting nutrition and guidance for the population. I'm not sure if it would be more convenient to consider the transportation system and health at the macro level, since citizens may not have much inference about this. Another point in the micro-environment would be schools and nurseries, which play a fundamental role in feeding the population of children and adolescents who live in the favelas...I don't know if it makes sense to think about this...but the other equipment and structure of the SUAS. Finally, perhaps incorporating social movements into third sector organizations, which often arise within communities and not as an external institution that is going to support a cause in the community.	considers any type of health service or unit that has direct contact with the individual, whether public or private (Basic Health Units, Private Practices, among others), and we added the variable "Health System" to the macroenvironment, which considers the infrastructure and set of health services that organize the health service in the microenvironment. We also included the variable "Social Assistance System" in the macro-environment, which considers social protection services for individuals, and "Social Services" in the micro-environment, which considers any type of social assistance service or unit that has direct contact with the individual, whether public or private. We consider public transport in the micro-environment because it is an important means of transport for individuals to move around, and we consider the transport system in the macro-environment, which is related to infrastructure and a set of services organized to allow people to move around efficiently and affordably within a city, metropolitan region or urban area. The description of local non-profit organizations and institutions includes social movements, and we have added examples to the description in the variable.

		In the microenvironment, we find Food Donation; what about the non-monetary exchanges and relationships to acquire food from the communities in the favelas? Could this somehow be incorporated into the model or is it already done by one of the variables?	In relation to the exchange of food items, we added the variable "Non-monetary food acquisition" to the model at the micro-environment level, which refers to the exchange of food items and food donations received.
		I believe that the "Water access" component can be added to the microenvironment.	We added the "Basic sanitation" variable to the model, which considers access to basic services such as drinking water distribution, sewage collection and treatment, urban drainage and solid waste collection.
		Thinking about the theoretical framework adopted, it seems to me that there was also a lack of connection with sustainability and environmental actions. I'm not sure how to explore this element in the communities...but I can see the emergence of collectives teaching cooking in the favelas, movements for uncomplicated and healthy vegetarian food, community kitchens, solar-powered stoves, sales of food considered to be out of the norm, public purchases of family farming for families registered with SUAS...in short...possibilities to think about as sustainability actions in these spaces that promote healthier eating...I think interviews with community leaders will help a lot on	We considered the model by Downs et al. (2020) in our references to think about and produce our model, however, we did not adapt it to take into account all the aspects and dimensions that the authors considered. Thus, sustainability was not included in this model as one of the factors affecting access to food by favela dwellers.

		this point.	
		I don't think it would be the case to make it much more complex, but having, for example, security and violence on the same level as e-commerce doesn't seem very clear to me.	Violence and the presence of e-commerce are factors in the environment that can affect access to food. The presence of e-commerce can favor access to food (online shopping with deliveries, without the need for residents to travel), and violence can be a factor that interferes with access to food (in a favela where there is conflict between factions, for example, shootings can occur, forcing residents to stay at home and not travel to buy food, or order food for delivery).
		It would be important, even in terms of measurement, to define examples of the microenvironment: would it be the family? the neighborhood? the school? the city? the favela itself, in this case?	The micro-environment refers to a context in which groups of people come together for specific purposes involving food, such as workplaces, schools, favelas and other community spaces.
		The microenvironment dimension is quite broad and could be better defined.	The micro-environment refers to a context in which groups of people come together for specific purposes involving food, such as workplaces, schools, favelas and other community spaces.
		The Microenvironment could include the variable Household Social Infrastructure: regular access to drinking water, electricity and garbage collection.	We added the "Basic sanitation" variable to the model, which considers access to basic services such as drinking water distribution, sewage collection and treatment, urban drainage and solid waste collection.

		As already mentioned... I have doubts about whether aspects related to regional eating habits will be included in the model. For example, a favela located in Rio de Janeiro may have a significant number of migrants from the northern region of the country, and the eating habits of this population may be related to their food choices within the favelas.	We added the variable "Food Culture" to the model at the individual level, which considers a set of ideas, beliefs, knowledge and practices transmitted over time, related to food and shared between and within groups. The geographical location of the favela was not included because we believe that this model is a general and broad model that can be applied to different contexts, and depending on the location and reality of the favela, one or more variables may not be included for the specific study of the location.
		Supply, prices and geographic location are determining factors in the purchase of food.	The concept of access to food that we adopted in the model considers:availability, physical proximity/accessibility, financial accessibility, acceptability, and convenience. Thus, the price of food is being considered in the model's outcome variable. Food supply is included in the variables on food establishments, street food sales, vegetable gardens, online food delivery platforms, e-commerce, and food and nutritional security equipment. The geographical location of the favela was not included because we believe that this model is a general and broad model that can be applied to different contexts, and depending on the location and reality of the favela, one or more variables may not be included for the specific study of the location.

		At the micro-environment level, the work-study variables could be separated out, since the study, in the case of schools, includes a public policy on access to food via school meals (PNAE). This could be important data for assessing access to healthy food via this public policy, or the lack of it.	The focus of this study was to assess the food environment of favela dwellers, and not specifically the organizational environment (such as school or work). Thus, when we consider schools, for example, we are considering the various forms of access to food that favela dwellers who attend schools may have, considering the food offered by the National School Feeding Program.
Macro-environment dimension	The macroenvironment definition adopted was proposed by Swinburn, Egger, & Raza in 1999 and refers to a group of industries, services, or supporting infrastructures that influence the foods consumed in various microenvironment contexts.	In addition, the "race/racism" component (Racism in the macro) should not be at the individual level, perhaps in the macro-environment, understanding and assuming racism as structural and structuring in Brazilian society, impacting everything from housing, in this case in favelas, to access to food.	The variable "Race or ethnicity" was kept at the individual level, and we added the variable "Racism" in the macro-environment, understanding its structural characteristic in society.
		I also think it will be important to assess whether other public welfare facilities and schools, for example, could be included. I thought of this because the element of security and violence came into play...	We included the variable "Social Assistance System" in the macro-environment, which considers social protection services for individuals, and the variable "Social Services" in the micro-environment, which considers any type of social assistance service or unit that has direct contact with the individual, whether public or private. With regard to schools, the focus of this study was to assess the food environment of favela dwellers, and not specifically the organizational environment (such as school or work). Thus, when we

			consider schools, for example, we are considering the various forms of access to food that favela dwellers who attend schools may have, considering the food offered by the National School Feeding Program.
		Companies and their sales strategies (advertising, marketing, placement in specific markets, etc.) must be clearly specified, as they are very important).	We included the "Marketing communication" variable in the macro-environment.
		Doubt about the elements included in the macro item, which have different orders of relationship with individuals, as I mentioned earlier.	The variables included in the macro-environment refer to a group of industries, services or support infrastructures that influence the food consumed in the various contexts existing in the micro-environment. For example, the food industry and its marketing strategies can influence the availability of food in different spaces in the microenvironment, such as schools, food outlets, e-commerce and online food delivery platforms.
		Another issue is that a conceptual model that takes into account the reality of the favelas in the Brazilian territory, in the macro-environment, would add the country's regions, taking into account that these territories have their own specific geographic characteristics, income distribution, public policy, etc.	The geographical location of the favela was not included because we believe that this model is a general and broad model that can be applied to different contexts, and depending on the location and reality of the favela, one or more variables may not be included for the specific study of the location.

Decision-making Level	The decision level definition was based on the concept proposed by Castro & Canella in 2022 when producing the conceptual model for the organizational food environment. In this context, decision level refers to power relations and decision-making processes that affect or interfere with the food environment, such as municipal, state, or national policies, programs, laws, and regulations.	I also think it would be interesting to include 'health policies' at the decision-making level.	We added the variable "Health services" to the model in the micro-environment, which considers any type of health service or unit that has direct contact with the individual, whether public or private (Basic Health Units, Private Practices, among others), and we added the variable "Health System" to the macroenvironment, which considers the infrastructure and set of health services that organize the health service in the microenvironment.
		I don't understand why the arrow indicating the decision-making level was created for public policies. I think they have to do with the macro-environment.	The decision-making level refers to the tools available in the environment for actions to be carried out in the service structures, which are described in the macro-environment. As an example, Food Supply Policies define the objectives, principles, guidelines and targets to be implemented by the National Food and Nutrition Security System and by the food production and distribution structures that exist in the macro and micro environments, such as public food and nutrition security facilities.
		Finally, I think it's worth including the rules on labeling and regulating food advertising in Brazil at the decision-making level.	The variable "Food regulatory agenda" was included at the decision-making level, which refers to labeling standards, regulation of food advertising, food taxation standards, among other regulatory provisions on food and nutrition.

		why do only public policies come into play at the decision-making level? would they be the only power relations present in the favelas? at the limit, it would also be important to consider the role of militias, conflicts between groups, and the presence of religious groups.	The decision-making level refers to the tools available in the environment for actions to be carried out in the service structures, which are described in the macro-environment. As an example, Food Supply Policies define the objectives, principles, guidelines and targets to be operationalized by the National Food and Nutrition Security System and by the food production and distribution structures that exist in the macro and micro environments, such as public food and nutrition security facilities. In order to include the role of criminal gangs and militias in the lives of favela residents, we included criminal organizations at the micro-environment level in the model through the variable "Other Organizations", which considers any other type of organization that may have some influence and openness in the favelas. With regard to religion, the model includes this variable at the individual level, due to its importance in the lives of families living in favelas and, consequently, in food consumption (restriction of certain types of meat, for example). In addition, the religious community can help in the donation and sharing of food.
		The decision-making level may give the impression that it is at the level of the individual. Changing the term, including	The decision-making level refers to the tools available in the environment for actions to be carried out in the service structures, which are

		who decides or changing the location of the arrow, not least because the decision-making level seems to influence the whole model.	described in the macro-environment. As an example, Food Supply Policies define the objectives, principles, guidelines and targets to be implemented by the National Food and Nutrition Security System and by the food production and distribution structures that exist in the macro and micro environments, such as public food and nutrition security facilities.
		The question of the decision-making level didn't seem very clear to me. And to think about these more structural elements of social assistance, in addition to FNS and donation equipment such as CRAS, CREAS, schools, UBS, etc? How can this be included in the model or not?	The decision-making level affects all the dimensions of the model (individual, micro-environment and macro-environment) and access to food. The graphical representation was adjusted to better represent the role of this level in the model. We included the variable "Social Assistance System" in the macro-environment, which considers social protection services for individuals, and the variable "Social Services" in the micro-environment, which considers any type of social assistance service or unit that has direct contact with the individual, whether public or private. In relation to food donations, we added a variable to the model at the micro-environment level "Non-monetary food acquisition", which refers to exchanges of food items and food donations received from any source.

		I was a little confused about the Decisional Level... in the final model, will it go through all the other levels or will it only be "linked" to Access to food. I suggest perhaps reviewing this "arrow".	The decision-making level affects all the dimensions of the model (individual, micro-environment and macro-environment) and access to food. The graphical representation has been adjusted to better represent the role of this level in the model.
		Isn't the decision-making level also part of the macro-environment in some way? Similarly, aren't some elements of the macro also part of the decision-making level?	The decision-making level refers to the tools available in the environment for actions to be carried out in the service structures, which are described in the macro-environment. As an example, Food Supply Policies define the objectives, principles, guidelines and targets to be implemented by the National Food and Nutrition Security System and by the food production and distribution structures that exist in the macro and micro environments, such as public food and nutrition security facilities.
		I wonder why the decision-making level is so close to the individual level. Some policies may have a more direct influence, but others not so much...	The decision-making level affects all the dimensions of the model (individual, micro-environment and macro-environment) and access to food. The graphical representation has been adjusted to better represent the role of this level in the model.
		In my opinion, the decision-making level should also relate to micro and macro structures. Since policies relate to public facilities, programs, FNS actions, social and	The decision-making level affects all the dimensions of the model (individual, micro-environment and macro-environment) and access to food. The graphical representation

		economic factors and also to all the food conglomerates and industries. Or it could have another term, in addition to decisional, since the way it is written, it is very much up to the individual to make the right decisions.	has been adjusted to better represent the role of this level in the model.
		Perhaps it's difficult to understand the Decisional Level, does it permeate the other levels? Is it a separate "category"? Does it relate only to access to food or does it cut across all the other levels?	The decision-making level affects all the dimensions of the model (individual, micro-environment and macro-environment) and access to food. The graphical representation has been adjusted to better represent the role of this level in the model.
		Isn't the decision-making level also part of the macro-environment in some way? Similarly, aren't some elements of the macro also part of the decision-making level? Seeing both dimensions together brought me this confusion and fusion that they have in some layers.	The decision-making level refers to the tools available in the environment for actions to be carried out in the service structures, which are described in the macro-environment. As an example, Food Supply Policies define the objectives, principles, guidelines and targets to be implemented by the National Food and Nutrition Security System and by the food production and distribution structures that exist in the macro and micro environments, such as public food and nutrition security facilities.
		The concept of the decision-making level is not so simple to understand.	We have updated the description to better describe this dimension. The decision-making level refers to the tools available in the environment for actions to be carried out in the service structures, which are described in

			the macro-environment. As an example, the Food Supply Policies define the objectives, principles, guidelines and targets to be implemented by the National Food and Nutrition Security System and by the food production and distribution structures that exist in the macro and micro environments, such as public food and nutrition security facilities.
		and "health policies" at the decision-making level.	We added the variable "Health services" to the model in the microenvironment, which considers any type of health service or unit that has direct contact with the individual, whether public or private (Basic Health Units, Private Practices, among others), and we added the variable "Health System" to the macroenvironment, which considers the infrastructure and set of health services that organize the health service in the microenvironment.
		The large corporations and power groups that operate in the food system, large industries, the media, and advertising and marketing agencies and departments are missing.	We included the variable "Food industry" in the macro-environment, understanding its role in modulating access to food, and we considered its marketing strategies in the description of this variable. And we included the variable "Other Organizations" in the micro-environment, which considers any other type of organization that may have some influence and openness in the favelas, such as

			actions carried out in these spaces to promote healthy eating coordinated by food industries.
		I understand that policies are at the decision-making level, but shouldn't state and municipal food and nutrition security plans be at the macro-environment level? I also think that SISAN should be highlighted, rather than just the policy, given that the policy is instituted by SISAN.	We included the variable "National, state, and municipal food and nutrition security plans" at the decision-making level, which refers to the goals to be implemented by the existing food production and distribution structures in the macro and micro environments, and the variable "National Food and Nutrition Security System" in the macro-environment, which refers to the structure that puts into practice the goals established by the Food and Nutrition Security Policy.
		As mentioned above, I suggest including food labeling and advertising regulations in the "decision-making" dimension.	The variable "Food regulatory agenda" was included at the decision-making level, which refers to labeling standards, regulation of food advertising, food taxation standards, among other regulatory provisions on food and nutrition.
		In the same way as the previous comment (Wouldn't the decision-making level also be part of the macro-environment in some way? Likewise, aren't some elements of the macro also part of the decision-making level?), THE CONCEPTS don't clarify what makes the difference between the dimensions essential, to say that an element is from one dimension, not another	The decision-making level affects all the dimensions of the model (individual, micro-environment and macro-environment) and access to food. The graphical representation has been adjusted to better represent the role of this level in the model.

		At the decision-making level and/or individual health status, consider the National Health Policy for the Black Population. At the decision-making or micro-environment level, in the item Public Food and Nutrition Security Equipment, include the PNAE and school meals as a food security strategy.	We added the variable "Health services" to the model in the microenvironment, which considers any type of health service or unit that has direct contact with the individual, whether public or private (Basic Health Units, Private Practices, among others), and we added the variable "Health System" to the macroenvironment, which considers the infrastructure and set of health services that organize the health service in the microenvironment. In relation to the National School Feeding Programme, the focus of this study was to assess the food environment of favela dwellers, and not specifically organizational environments such as schools. Thus, when we consider schools, we are considering the various forms of access to food that favela dwellers who attend schools may have, considering the food offered by the National School Feeding Program.
		The decision-making level could include variable educational policies. I believe it could be a good parameter for identifying the lack of coverage of the subject in the school curriculum, even though it is a cross-cutting theme in the BNCC. We need to demand educational policies that include EAN.	The focus of this study was to assess the food environment of favela dwellers and its impact on food access, and not specifically organizational environments such as schools. In this way, when we consider schools, we are considering the various forms of access to food that favela dwellers who attend schools may have. The subject of the school curriculum is a specific aspect to be further

			developed in models on the organizational environment, or specifically on the school food environment.
Individual Level			
Gender	Socially constructed characteristics, roles, behaviors, expectations, and identities associated with being male, female, or other gender identities such as non-binary, agender, etc.	I think we need to review the definitions of gender identity and racism.	With regard to the "Gender" variable, its definition states that we consider all gender identities. In relation to racism, we added this variable to the macro-environment, understanding its structural characteristic in Brazilian society.
Age	The life stage of the individual, expressed by the amount of time elapsed since birth. Example: childhood, adolescence, senescence.	-	-
Income	The financial importance that the individual periodically receives as remuneration for work or services rendered. Examples: salary, pension, aid, among other financial resources.	-	-
Race and racism	The belief that it is possible to classify humans into different naturally hierarchical races based not only on physical aspects but also on moral, psychological, intellectual, and cultural characteristics.	Specifically on race and racism, we need to correct the idea of biological race. I think it's enough to use the term racism. Racism is not an individual expression, so racism as a structure shapes the entire food environment, and is part of the macro and micro environment, shaping public policies and urban spaces themselves.	The variable "Race or ethnicity" was kept at the individual level, and we added the variable "Racism" in the macro-environment, understanding its structural characteristic in society.
		Include education and remove race and include racism at other levels.	The variable "Race or ethnicity" was kept at the individual level, and we added the variable

			"Racism" at the macro-environment level, understanding its structural characteristic in society. With regard to schooling, we added a variable to the model at the individual level, "Socioeconomic status", which takes into account income (of any kind, salary and benefits) and schooling.
		Regarding the "Race/racism" component: I thought it was very important to consider this aspect in the conceptual model, but I missed the fact that, throughout the text described, there was a contextualization of these issues. If you are considering that race/racism are important factors in this study, then we need to talk about why this is so and about the composition of these favela territories, which are mostly inhabited by black people. So I think a brief contextualization of this historical process that explains why these people occupy these spaces is in order.	The variable "Race or ethnicity" was kept at the individual level, and we added the variable "Racism" in the macro-environment, understanding its structural characteristic in society.
		The description of the variable "race and racism" seems confusing to me in two respects: 1. Race is a belief from a biological point of view, but from a sociological point of view it is a material reality. Treating it as a "belief" without making this distinction can lead to misinterpretations; 2. I understand that individuals will be classified	The variable "Race or ethnicity" was kept at the individual level, and we added the variable "Racism" in the macro-environment, understanding its structural characteristic in society.

		based on their self-declared race. Racism is a power relationship structured around the hegemony of power of one race over the other(s). In Brazil, it is expressed through the accumulation of privileges by individuals of the white race to the detriment of individuals of other races. Thus, racism may appear (I believe it will) in the analysis of the results, but it is not a variable in itself.	
		The description offered in the variable "Race and racism" only details what racism is and not race.	The variable "Race or ethnicity" was kept at the individual level and its description updated to the ethnic or racial denomination of people in Brazil, which includes the terms black, brown, yellow, indigenous or white, and we added the variable "Racism" in the macro-environment, understanding its structural characteristic in society.
		At the individual level, it would be important to pay attention to the item RACE/COLOR/RACISM, considering the data from the last IBGE census and the data from the II VIGISA survey, the majority of the population living in favelas are black people. This will have an impact on social and cultural relations with food and eating, and even on the concept of healthy eating, for example.	The variable "Race or ethnicity" was kept at the individual level, and we added the variable "Racism" in the macro-environment, understanding its structural characteristic in society.
Religion	A set of principles, beliefs, and practices of religious	Regarding the "religion" variable - not all	We have updated the definition of the variable

	doctrines based on sacred texts that unite their followers into the same moral community.	religions are based on sacred books. In Afro-Brazilian religions such as Candomblé and Umbanda, for example, religious knowledge is transferred orally.	"Religion" to: a set of beliefs and social practices related to the notion of the sacred, which bring together all those who adhere to it in the same moral community.
Working or studying hours	The daily period when the resident is available for work and/or is in class or dedicating time to studies. It also includes travel time to these locations when activities are conducted in person.	-	-
Health Status	The health conditions of the resident that allow or prevent access to food. Examples: comorbidities that affect mobility or the development of daily activities such as obesity, chronic non-communicable diseases (NCDs), congenital and/or acquired physical and/or mental disabilities that affect or hinder mobility or the development of daily activities.	The term "state of health" includes illnesses and disabilities, and I think it's important to separate them. People with disabilities cannot necessarily have/be in poor health. In fact, certain disabilities, such as motor or visual impairments, can influence the individual's relationship with the food environment in which they live.	The health status variable was updated and subdivided into two: "Health status" and "Disabilities", as these are factors that can affect displacement in order to access food.
		As mentioned above, I suggest separating diseases and disabilities within the health status variable.	The health status variable was updated and subdivided into two: "Health status" and "Disabilities", as these are factors that can affect displacement in order to access food.
Food production or animal rearing	The production of food and/or raising animals for personal consumption, with the possibility of selling the surplus in the region. Examples: gardens (vertical, horizontal) in backyards or balconies of residences, or raising animals for food purposes (as well as their products such as milk, honey, and eggs).	As I said earlier, I think it's worth changing the term from the element of food production and/or animal husbandry to food production and/or animal husbandry for subsistence.	The variable on "Food production or animal rearing" takes into account the food that families living in favelas produce for their own consumption, barter and the sale of surpluses (which can help the family's income and, consequently, the purchase of food).
		And the item food production in the individual field, in the conceptual model,	The variable on "Food production or animal rearing" takes into account the food that

		must be specified that it is basically for self-consumption, subsistence, otherwise it seems that it is the food system as a whole.	families living in favelas produce for their own consumption, barter and the sale of surpluses (which can help the family's income and, consequently, the purchase of food).
		In food production, I suggest including examples of the production of meals or preparations, such as marmitas, cakes, sacolé/dindim, homemade sweets, which are very common in favela households. I think this would be at the micro-environment level. In the options you have, I haven't explicitly identified examples of this and I think it's very common, so it would be interesting to make it more explicit.	The variable on "Food production or animal rearing" considers the food that families living in favelas produce for their own consumption, bartering and selling surpluses (which can help with the family's income and, consequently, the acquisition of food). The production of meals or other types of culinary preparation for sale is not included in the "Food production or animal rearing" variable, as this type of production could be included in forms of income acquisition.
		Also at the individual level, consider Food Production and/or Animal Husbandry, non-colonized food systems and non-colonized food plants, taking into account the racial and cultural identity of the peripheral population (Matos de Comer, Plantas da negritude, comida de santo, comida de terreiro). Reference authors on the subject are Bruna Thomas de Oliveira Pedrosos, Renata Sirimarco, Lourence Crirstina Alves, Antônio Bispo dos Santos.	The variable on "Food production or animal rearing" considers all the food that families living in favelas can produce for their own consumption, bartering and selling surpluses.
Food	Data, knowledge, messages, and content related to food, nutrition, diets, eating habits, food security, and all	In addition to this, I suggest changing the term information on food and nutrition to	In relation to information about food and/or eating, we added the variable "Food literacy"

Information	aspects involving food intake.	access to adequate education and knowledge, since these social factors will influence and determine the understanding of data and information on food and adequate and healthy nutrition.	at the individual level, which refers to the resident's ability to obtain, process and understand information about nutrition, and we included the variable "Food Information" at the micro-environment level, which refers to the information available in the environment about food, such as nutritional tables and lists of ingredients, food advertising, nutritional claims, among other information.
		I think that information about food and/or eating includes two perspectives: information given on the product or by the place and the level of prior information that the individual has, so my suggestion is to divide these two dimensions of knowledge: 1. Leaving the individual with the term literacy in nutrition or health since it is related to the individual's level of knowledge and ability to understand and 2. Availability of information at the micro-environment level. I think that violence would also come under the individual level, thinking of issues where the neighborhood is okay, but there is domestic violence, for example.	In relation to information about food and/or eating, we added the variable "Food literacy" at the individual level, which refers to the resident's ability to obtain, process and understand information about nutrition, and we included the variable "Food Information" at the micro-environment level, which refers to the information available in the environment about food, such as nutritional tables and lists of ingredients, food advertising, nutritional claims, among other information.
		In addition, I suggest changing the variable information about food and/or eating to access to quality education and knowledge,	In relation to information about food and/or eating, we added the variable "Nutritional literacy" at the individual level, which refers to

		because this is individually what will affect the understanding of the information received and consequently habits and appropriate and healthy choices. And so, the element of information should be included in the micro field as factors that influence access to adequate and healthy food.	the resident's ability to obtain, process and understand information about nutrition, and we included the variable "Information about food" at the micro-environment level, which refers to the information available in the environment about food, such as nutritional tables and lists of ingredients, food advertising, nutritional claims, among other information.
Commute	Time spent and route taken when traveling between different physical points using any means of transport.	-	-
Micro-environment Level			
Community and neighborhood vegetable gardens	Presence of community gardens or productive backyards of neighbors or other forms of food production or animal husbandry for distribution and sale in the community. Examples: private gardens (vertical, horizontal) in the backyards or balconies of residences and/or community gardens initiated by the population or the government in public spaces.<br>Donation of food on a small or large scale by a single person, volunteer groups, or organizations and institutions, which may or may not be mediated by third sector organizations or community leaders. These donations can be made by churches, non-governmental organizations, government institutions, among other associations or individuals who donate food.	Evaluate whether it is worth using the term urban agriculture, in order to dialog with actors who consider that the term gardens gives the impression that they are only places with low production and for self-consumption...	The variable "Community and neighborhood vegetable gardens" takes into account any type of place where food is produced in favela areas or where people living in favelas go to buy or acquire (free of charge or through barter) food.
		#include agroecological fairs	The variable "Community and neighborhood vegetable gardens" takes into account any type of place where food is produced in favela areas or where people living in favelas go to buy or acquire food (free of charge or on a barter basis). We added the variable "Food fairs" to the microenvironment, which considers physical spaces for food sales in fixed locations that use tents and collapsible equipment that can offer *fresh* and minimally

			processed foods, culinary preparations, among other types of food.
Food Donation	Donation of food on a small or large scale by a single person, volunteer groups, or organizations and institutions, which may or may not be mediated by third sector organizations or community leaders. These donations can be made by churches, non-governmental organizations, government institutions, among other associations or individuals who donate food.	In the micro-environment, we find Food Donation; what about the non-monetary exchanges and relationships to acquire food from the communities in the favelas? Could this somehow be incorporated into the model or is it already done by one of the variables?	In relation to the exchange of food items, we added a variable to the model at the micro-environment level "Non-monetary food acquisition", which refers to the exchange of food items and food donations received from any source.
Formal food stores	Establishments with formal fixed points that pay taxes, are inspected, and have government registrations focusing on food sales. These food sale establishments have a National Register of Legal Entities (CNPJ) or other types of registration for regulation.	-	-
Informal Food stores	Establishments with informal fixed points focusing on food sales, which may or may not be regulated and inspected by government agencies, characterized by lack of specialization, low capital investment, non-reporting, non-payment of some or all taxes, and social innovations. These food sale establishments do not have a National Register of Legal Entities (CNPJ) or other type of registration but can be registered with the National Health Surveillance Agency (Anvisa) for sanitary inspections.	Definition of the informal food environment Considering the scarcity of studies on the informal, I think that in this model it is worth problematizing which concept of the informal environment will be used. For example, some articles only consider those that are not regulated by government bodies. I think it's worth problematizing the challenge of the concept. For example: the street vendor can be registered with the PBH, the mate seller on the beach in Rio too... and because he is regulated he would be more formal? Can we compare them to supermarket owners? He loses one of the	To define formality in this model, we consider any type of registration and inspection to which establishments are subject, whether state or municipal, such as the National Register of Legal Entities (CNPJ) or municipal unions that charge fees to regularize the establishment. In addition, there are the health inspections carried out by the municipalities, which mostly occur through complaints (not following tax registration lists). Thus, informal sales establishments are establishments with fixed points, which may or may not be subject to inspection by the health inspectorate, but do not pay fees. Examples

		elements of formality (he's regulated), but he's still exposed to other vulnerable issues. Ambikapathi et al., 2021 - ''The study team conducted a photographic survey of all the different types of food vendors in the study area and then held discussions to decide how vendors can be categorized to improve the reliability of the ratings; these were (1) physical infrastructure and (2) consistent daily location. Those with permanent physical infrastructure such as cement-built stores that were consistently present in the same locations were marked as formal suppliers. Vendors with semi-permanent structures (e.g. wooden stalls and umbrellas) but who maintained consistent daily locations were categorized as semi-formal vendors. Mobile vendors who walked or used bicycles or carts were categorized as informal vendors.'' Downs, used here in this model doesn't make it very clear - 'Informal food environments are those that are often not regulated through formal governance structures.	include the sale of food in garages and on doorsteps, or even signs on doorsteps informing people that some kind of culinary preparation is coming. Street food sales are described in another variable, "Street vending", which considers mobile types of food sales, which can be formal (paying municipal or state taxes) or informal (not paying taxes). Tents and fairs are included in the variable "Food fairs", which was included in the micro-environment, which considers physical spaces for the sale of food in fixed locations using tents and collapsible equipment that can offer fresh and minimally processed food, culinary preparations, among other types of food.
		Adjust text from "Informal Food Sales Establishments" to: These food sales establishments do not have a National Register of Legal Entities (CNPJ) or any other type of registration, but they do have the	To define formality in this model, we consider any type of registration and inspection to which establishments are subject, whether state or municipal, such as the National Register of Legal Entities (CNPJ) or municipal

		possibility of being registered with the National Health Surveillance Agency (Anvisa) for health inspections.	unions that charge fees to regularize the establishment.
Public Food and nutrition security facilities	Equipment that integrates a set of policies and actions implemented by the state to guarantee the human right to adequate and healthy food for the entire population. These are physical structures, programs, or services offered by the government at different levels (municipal, state, or federal) to promote access to quality food, adequate nutrition, and food security. Examples: popular restaurants, open markets, food banks, public markets, community kitchens, among others.	-	-
Street vending	The sale of food in open or closed spaces permanently with a fixed point or not. The street vendor may or may not be registered with government agencies and pay taxes, being subject to inspection. Examples: food vendors with a mobile selling space (carts, mobile stands, cars, bicycles, among others).	Informal AA and street vendors are separate - in the paper by Medina et al. 2022, street vendors are part of the informal environment. ''Informal food outlets also include mobile outlets that sell food from a mobile vehicle, such as a truck, wagon, trailer, kiosk or stall''. Why separate the street vendor from the informal environment?	To define formality in this model, we consider any type of registration and inspection to which establishments are subject, whether state or municipal, such as the National Register of Legal Entities (CNPJ) or municipal unions that charge fees to regularize the establishment. In addition, there are the health inspections carried out by the municipalities, which mostly occur through complaints (not following tax registration lists). Thus, informal sales establishments are establishments with fixed points, which may or may not be subject to inspection by the health inspectorate, but do not pay fees. Examples include the sale of food in garages and on doorsteps, or even signs on doorsteps informing people that some kind of culinary

			preparation is coming. Street vending is described in another variable, "Street vending", which considers mobile types of food vending, which can be formal (paying municipal or state taxes) or informal (not paying taxes). This variable structure was developed with Brazil's reality in mind and the different forms of access to food.
Food E-commerce	Sale of food through online stores where all parts of the product purchase are online: product selection, choice of delivery or pick-up address, payment method, and purchase.	Considering the Food model's definition of e-commerce, where would the physical store that also has e-commerce available (with, for example, on-site pick-up) come in? Does it also come under this definition?	If the in-person establishment has the option to buy online, it also falls into the e-commerce category (in addition to that of food outlets).
		"Physical proximity or accessibility" should include e-commerce, since food goes to people these days.	E-commerce is defined by the sale of food through virtual stores on the internet, where all parts of the purchase of the product are online: the selection of the product, choice of address for delivery or collection, form of payment and purchase.
Online food delivery platforms	Applications that host one or more companies that sell ready-to-eat or non-ready-to-eat food with a delivery service. They are business platforms offering order management, delivery, and payment services to food-selling establishments.	-	-
Local non-profit organizations and institutions	Non-profit organizations that provide free support and services to favela residents.	I would adjust the text under "Local Third Sector Organizations and Institutions" (change third sector to non-profit) to: Non-profit organizations, including public	We adjusted the text of the variable. We also included the variable "Other Organizations" in the micro-environment, which considers any other type of organization that may have some

		interest civil society organizations, that provide free support and services to favela dwellers.	influence and openness in the favelas.
		Local Third Sector Organizations and Institutions - I suggest citing examples	We adjusted the text of the variable, which includes, for example, the Central Única de Favelas. We also included the variable "Other Organizations" in the micro-environment, which considers any other type of organization that may have some influence and openness in the favelas, such as criminal factions.
Internet access	Availability of broadband connection or wireless internet access points, either private or free.	-	-
Security and Violence	Perception of safety and violence experienced by residents influenced by drug trafficking, policing, confrontations, assaults, among other types of situations causing insecurity or safety to residents.	to think about the weight of conflicts and disputes between drug trafficking groups and militias (not if this is already covered at the micro-environment level, in the security category	We updated the description of the "Security and Violence" variable to add possible factors that could interfere with this aspect. We also included the variable "Other Organizations" in the microenvironment, which considers any other type of organization that may have some influence and openness in the favelas, such as criminal factions and militias.
Alternative transportation	Existence of unregulated collective and/or individual transportation means for travel within the favela. Examples: motorcycle taxis, vans, among others.	Public and alternative transportation are part of the micro-environment. What about personal transportation? Like car, bicycle... individual level?	Personal transportation is being considered in the "Commute" variable. Commuting refers to the individual dynamics that favela residents need to organize and plan in order to move around the favela and the city, considering commuting to work, study, shopping, social life, among others. This commute can be on foot, using personal, alternative or public

			transport, depending on each individual's routine and organization, as well as the availability of alternative or public transport in the micro-environment.
Public transportation	Existence of accessible public transport and lines that access favelas and connect to other parts of the city.	-	-
Work or Study Place	Food sold and/or provided free of charge for immediate consumption in educational institutions and workplaces located in favelas or frequented by favela residents.	-	-
Walkability	The physical structure of the urban space that allows and encourages walking. Examples: street elevation level, existence and condition of sidewalks, sidewalk width, obstructions, public lighting, tree coverage, crosswalks, existence and access to bus stops, block size, among others.	I don't think the term "walkability" is so clear from just looking at the model, but it is well described in the detailed table.	Walkability is defined by the Institute for Transport Policy and Development as the extent to which the characteristics of the urban environment favor its use for walking.
Macro-environment Level			
Marketing communication	Set of strategies, messages, and marketing practices used by companies and organizations in the food industry to promote and sell their food products. These strategies aim to influence consumer perception, increase brand recognition, and stimulate food product purchases. Examples: television and social media advertising, attractive labels with health claims, reward programs, combos, and discounts, among others.	-	-
Master plan and municipal strategic planning	Legal and technical urban planning tool used by municipalities and municipal authorities to guide urban development and land use planning. The master plan is a document that establishes guidelines, objectives, policies,	I missed the City's Strategic Plan in addition to the Master Plan	We have updated the variable description to urban planning and management tools used by cities and authorities to guide urban development and planning.

	and norms for the growth and land use of a city or metropolitan region.		
Food Production and Distribution	Interconnected processes, activities, and infrastructures involved in food production and distribution, including food cultivation, the transformation of raw materials into food products, and the physical movement of food from production to sale points or consumers.	-	-
Public transportation system	Infrastructure and set of organized services to enable the efficient and accessible movement of people within a city, metropolitan region, or urban area. It is designed to meet the mobility needs of urban populations.	-	-
Decision-making Level			
Food and nutrition policies and Food and nutrition security policies	Sets of strategies, plans, programs, and actions implemented by governments and organizations to promote healthy eating, ensure adequate and sufficient access to healthy foods, and prevent malnutrition and food and nutrition insecurity. These policies are designed to address issues related to nutrition, public health, food security, and access to healthy and sustainable foods.	-	The variable was divided into "Food and nutrition policies" and "Food and nutrition security policies" after the authors considered the significance of each set of policies.
Social Policies	Set of actions, programs, measures, and strategies implemented by the state or other governmental and non-governmental institutions to address social issues and promote the well-being of the population. These policies aim to meet basic needs and ensure fundamental rights for individuals, especially those in situations of vulnerability or social disadvantage.	-	-
Food Supply Policies	Set of strategies, regulations, governmental actions, and programs aimed at ensuring an adequate and sustainable	-	-

	food supply for the population of a country, region, or community. The main objective of these policies is to ensure that the population has access to safe, nutritious, and sufficient food to meet their basic dietary needs.		
Public Security Policies	Set of strategies, guidelines, actions, and measures implemented by governments and public institutions with the objective of promoting security, preventing crime, protecting citizens, and ensuring the enforcement of laws within a given jurisdiction. These policies are designed to address a variety of security-related challenges, ranging from crime prevention to emergency and disaster response.	-	-
Graphic representation			
-	-	I like the format of overlapping circles, maybe the arrow of the decision-making level shouldn't only focus on access...for example, public safety policies don't act directly on access, but rather on the micro-environment that will allow more businesses to be present in the community, guaranteeing walkability through the neighborhood. Perhaps we need to rethink how the decision-making level fits into the macro and micro scenarios and into access...it acts in three different ways.	The decision-making level affects all the dimensions of the model (individual, micro-environment and macro-environment) and access to food. The graphical representation has been adjusted to better represent the role of this level in the model.
-	-	I believe that the arrow format of the decision-making level places too much emphasis on it, as if it were more important	The decision-making level affects all the dimensions of the model (individual, micro-environment and macro-environment) and

		than the others. I suggest placing it as a rectangle in the background, more comprehensive than the macro-environment level (it is the "backdrop").	access to food. The graphical representation has been adjusted to better represent the role of this level in the model.
-	-	The decision-making level influences all the other levels and the way it's put seems to influence access to food on a daily basis without being related to the other levels	The decision-making level affects all the dimensions of the model (individual, micro-environment and macro-environment) and access to food. The graphical representation has been adjusted to better represent the role of this level in the model.
-	-	I didn't particularly find the graphic didactic.	The graphical representation was adjusted to better represent the role of each domain in the model.
-	-	Reflect better on the decision-making level	The decision-making level affects all the dimensions of the model (individual, micro-environment and macro-environment) and access to food. The graphical representation has been adjusted to better represent the role of this level in the model.
-	-	The graphic representation of the microenvironment lacked the element of food advertising.	The food advertising and publicity element is now included in the "Marketing Communication" variable.
-	-	I think the graph gives a good picture of the points that interact with food access. But I also think about how some people can understand/interpret individual issues and disassociate the whole.	The graphical representation was adjusted to better represent the role of each domain in the model.
-	-	Because we're talking about food from the	The graphic representation has been adjusted

		favela, I think that it could then be made available for more people to access, so this table could be more didactic. It could have elements, visuals, after all we also want to communicate with those who don't read, or who don't read as much, but if it's just for academia, this is the language.	to better represent the role of each domain in the model, with the aim of being didactic for readers.
-	-	I made an earlier comment about the decision-making level, but it was more about graphic visualization than understanding. However, the way I understood it I think it should be visually more encompassing of the other 3 dimensions.	The decision-making level affects all the dimensions of the model (individual, micro-environment and macro-environment) and access to food. The graphical representation has been adjusted to better represent the role of this level in the model.
-	-	This will depend on the conception of the subjects in each favela, as this type of political training is not the reality of all favelas and peripheries	We consider this to be a general and broad model that can be applied to different contexts, and depending on the location and reality of the favela, one or more variables may not be included for the specific study of the location.
