# Supplementary material for: Conceptual Model on Access to Food in the Favela Food Environment
Source: Int J Environ Res Public Health. 2024 Oct 26;21(11):1422. doi: 10.3390/ijerph21111422 (PMC11593643; doi:10.3390/ijerph21111422)

Supplementary Material S4. Graphical Representation of the Final Version of the Conceptual Model

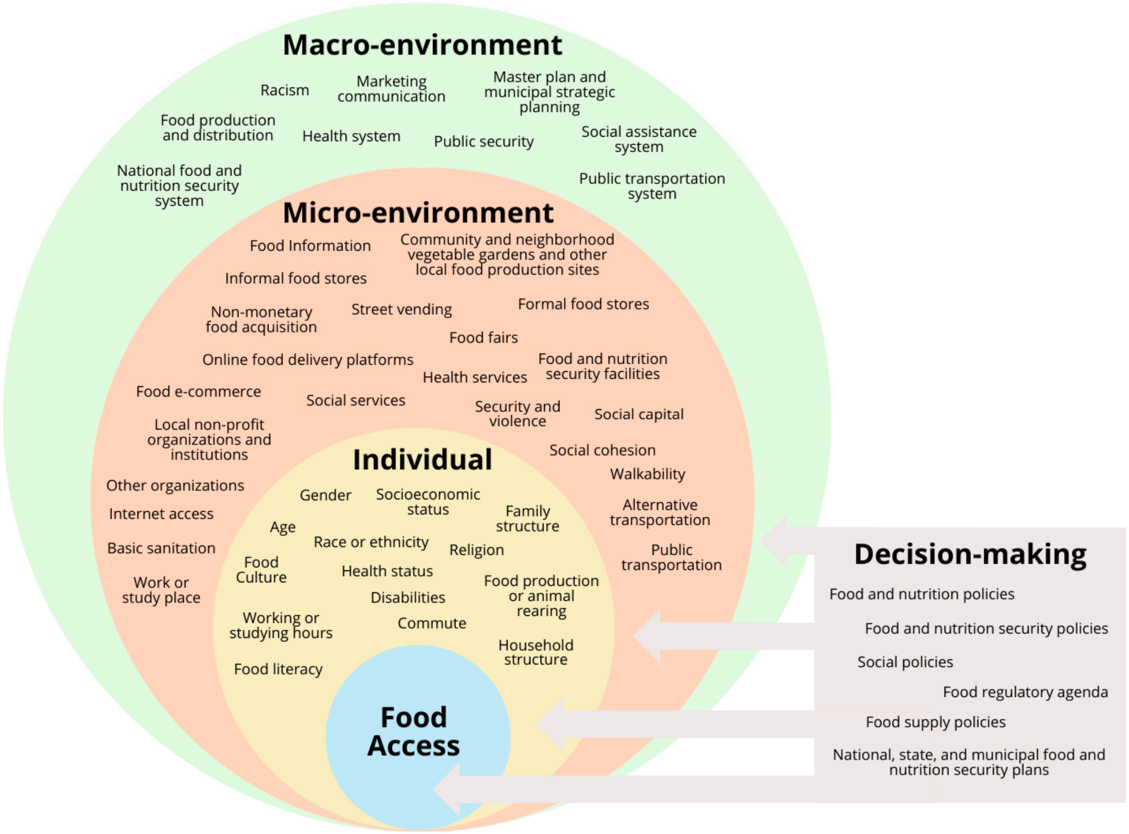

Supplement: Supplementary file 1 [file ijerph-21-01422-s001.zip › Supplementary Material S4.pdf]
